# Preoperative Diagnostic Value of Spectral CT for Predicting Perineural Invasion in Esophageal Cancer

**DOI:** 10.1002/cam4.71403

**Published:** 2025-11-20

**Authors:** Zongbo Li, Wenzheng Lu, Yiheng Zhou, Xiaofei Wu, Yuxi Ge, Wei Shao, Shudong Hu

**Affiliations:** ^1^ Department of Radiology Affiliated Hospital, Jiangnan University Wuxi Jiangsu China

**Keywords:** esophageal cancer, nomogram, perineural invasion, spectral CT

## Abstract

**Purpose:**

To assess the diagnostic value of preoperative spectral CT quantitative parameters in predicting perineural invasion (PNI) in esophageal squamous cell carcinoma (ESCC), which is a critical prognostic factor associated with increased recurrence and poor survival. Preoperative identification of PNI can guide individualized treatment strategies.

**Methods:**

A retrospective analysis was conducted on 78 patients with EC who underwent preoperative spectral CT. Patients were classified into PNI‐positive and ‐negative groups on the basis of histopathological findings. Spectral CT parameters, including conventional single‐energy CT value (Sect), virtual monochromatic images, effective atomic number (Zeff), and iodine concentration (IC), were compared between groups. Statistical analyses were performed through t, rank sum, and chi‐squared tests. A diagnostic nomogram was constructed by employing independent predictors and validated via receiver operating characteristic curve analysis with DeLong's test for the pairwise comparison of the area under the curve (AUC), ensuring the robust evaluation of discriminative performance.

**Results:**

Significant differences in spectral CT parameters were observed between the PNI‐positive and PNI‐negative groups. Specifically, the PNI‐positive group exhibited higher values of 40–70 keV, Zeff, and IC (all *p* < 0.05) than the PNI‐negative group. Among parameters, 40 keV demonstrated the highest predictive accuracy for PNI, with an AUC of 0.943. Binary logistic regression identified CYF, Sect, and 40 keV as independent predictors of PNI status. A nomogram incorporating these variables achieved the optimal diagnostic performance with an AUC of 0.971.

**Conclusion:**

Preoperative spectral CT quantitative parameters, particularly 40 keV, Zeff, and IC, provide valuable insights for assessing PNI in ESCC. The integration of spectral CT parameters with clinical features can significantly improve the accuracy of PNI diagnosis.

**Abbreviations**
AUC: area under the curve
CA199: carbohydrate antigen 199
CI: confidence interval
CYF: cytokeratin fragment 21‐1
ESCC: esophageal squamous cell carcinoma
IC: iodine concentration
ICC: intraclass correlation coefficient
PNI: perineural invasion
Sect: conventional single‐energy CT
Zeff: effective atomic number

## 1 Introduction

Esophageal squamous cell carcinoma (ESCC) is one of the most prevalent and lethal malignant tumors worldwide, and most patients with this malignancy are diagnosed at an advanced stage with a poor prognosis [1]. Perineural invasion (PNI), a key indicator of tumor aggressiveness, is remarkably associated with a high recurrence rate and low survival rate after surgery [2]. It is characterized by the infiltration of cancer cells along the peripheral nerve sheaths of the esophagus and often leads to accelerated disease progression and increased therapeutic complexity [3]. Therefore, the precise preoperative identification of PNI is essential for developing individualized treatment strategies and improving patient prognosis [4].

Conventional PNI assessment mostly relies on invasive pathology or is limited by the resolution of conventional imaging techniques [5]. In recent years, energy spectral CT (spectral CT) has provided new ideas for the noninvasive assessment of PNI by analyzing tissue components through multienergy imaging. This technique can generate virtual single‐energy images and virtual noncontrast, effective atomic number, and iodine concentration (IC) parameters, which substantially improve tissue contrast and feature resolution and provide potential biomarkers for quantifying tumor aggressiveness [6, 7, 8].

Studies have confirmed the value of conventional (single‐energy) CT for the prediction of PNI status in ESCC, and spectral CT technology has also been applied in the assessment of PNI in patients with other malignant tumors, such as gastric cancer, head and neck tumors, and colorectal cancer [9]. In the field of ESCC, spectral CT has been widely used for preoperative multidimensional assessment, including the characterization of primary foci, prediction of vascular invasion and lymph node metastasis, and monitoring of neoadjuvant therapy efficacy [10]. However, no reports on the use of spectral CT quantitative parameters for the preoperative prediction of esophageal PNI status exist. Therefore, this study aims to investigate the efficacy of preoperative spectral CT quantitative parameters for the prediction of PNI status in patients with ESCC and thus provide a noninvasive and accurate preoperative assessment tool for clinical use.

## 2 Materials and Methods

### 2.1 Patients

This retrospective study was approved by our institutional ethics review board. Written informed consent was waived. Between May 2023 and July 2024, 112 patients with histologically confirmed ESCC were enrolled in this study. The inclusion criteria were (1) patients with a histopathological diagnosis of ESCC and (2) who underwent spectral CT imaging within two weeks prior to surgery. The exclusion criteria were (1) patients with pathological results lacking PNI status (*n* = 3); (2) lesions with a minimum diameter < 5 mm (*n* = 14); (3) patients who received preoperative radiation therapy or chemotherapy (*n* = 13); and (4) poor lesion image quality due to artifacts (*n* = 4). Ultimately, 29 PNI‐positive patients and 49 PNI‐negative patients were included in this study. Figure S1 presents a flowchart illustrating patient selection.

### 2.2 Spectral CT Scan

All patients underwent spectral CT (IQ on Spectral CT, Philips, version 4.7.5), including plain and enhanced chest scans. Imaging parameters were set as follows: tube voltage of 120 kV peak; automatic modulation of tube current; pitch of 0.99; rotation time of 0.75 s; detector collimation of 64 mm × 0.625 mm; and layer thickness of 1.25 mm. Enhancement scans were performed 70 s after the injection of a nonionic contrast agent (iodixanol, 300 mg iodine/mL; GE Healthcare, the USA). The contrast agent was injected through a peripheral vein in the anterior fossa of the elbow by using an automated high‐pressure syringe at a rate of 3.0 mL/s (dose: 1.2 mL/kg) and was followed by the injection of 30 mL of saline at the same rate for washout.

### 2.3 Image Evaluation

Spectral CT images were independently postprocessed and analyzed by using Spectral CT Viewer software on IntelliSpace Portal 10 (Philips Healthcare). The spectral image series included virtual monoenergetic images, as well as spectral profile, effective atomic number (Zeff), and IC plots. Two radiologists (readers 1 and 2) with 5 and 10 years of experience in chest imaging performed the study. The quantitative CT parameters of lesions were measured independently without histologic information. The region of interest was circled along margins to maximize the extent of lesions on the transverse section while avoiding visible areas of necrosis. The quantitative values of lesions were obtained (40–70 keV attenuation, Conventional single‐energy CT [Sect], IC, and Zeff). In addition, two radiologists evaluated Sect imaging features (including tumor length, clinical T‐stage [cT‐stage], and clinical N‐stage [cN‐stage]). Tumor length in the craniocaudal direction was measured manually on sagittal images with reference to cross‐sectional and coronal projections.

### 2.4 Pathological Evaluation

Postoperative pathological evaluation was performed by a senior pathologist with 10 years of clinical experience in thoracic oncology and who had no knowledge of CT images and clinical information. Patients with tumor cells present within neural structures and spreading along nerve sheaths were included in the PNI‐positive group, whereas those without PNI were included in the PNI‐negative group. Histological grading was divided into poorly, moderately, and highly differentiated. Staging was performed in accordance with the criteria of the American Joint Committee on Cancer Tumor–Node–Metastasis Classification of Malignant Tumors, eighth edition. Clinical data, including age, gender, carbohydrate antigen 199 (CA199), and cytokeratin fragment 21‐1 (CYF), were collected from digital medical records.

### 2.5 Statistical Analysis

All statistical analyses were performed by using SPSS 26.0 and R 4.2.2 software. Normality distribution was assessed by using the kurtosis‐skewness test. Quantitative variables were expressed in the form of mean ± standard deviation or median (interquartile range), and categorical variables were expressed in the form of numbers and percentages in accordance with the normality of their distribution. The t or rank sum test was used for quantitative variables, and the chi‐squared test was employed for categorical variables. Intraclass correlation coefficient (ICC) was applied to evaluate the interobserver agreement of the spectral CT–derived quantitative parameters. Diagnostic efficacy was evaluated through subject work characteristic (receiver operating characteristic) curve analysis, and thresholds were determined on the basis of the Jordon index. The area under the curve (AUC) was compared by using the DeLong test. Bonferroni's method was utilized to interpret multiple comparisons. Binary logistic regression analysis was applied to determine the independent predictors of the quantitative parameters of spectral CT and clinical characteristics, with *p* < 0.05 as the independent predictor. Subsequently, a nomogram was constructed to predict PNI status on the basis of the results of binary logistic regression. Calibration and decision curves were then plotted to assess the clinical utility of the predictive model. *p* < 0.05 was considered statistically significant.

## 3 Results

### 3.1 Patient Characteristics

A total of 78 patients (62 males and 16 females; median age, 68 years) were included in this study (Table 1). No significant differences were observed between the PNI‐positive and ‐negative groups in terms of age and gender (both *p* > 0.05). However, tumor length, cT‐stage, cN‐stage, histological grade, CA199, and CYF differed significantly between the two groups (all *p* < 0.05).

**TABLE 1:** Clinical characteristics of the ESCC patients.

Characteristics	Overall, *n* = 78	PNI		*p*
		Positive (+) *n* = 29	Negative (−) *n* = 49	
Age, mean ± SD	68.19 ± 9.52	67.24 ± 8.07	68.76 ± 10.32	0.501
Gender, *n* (%)				
Male	62 (79.49)	21 (72.41)	41 (83.67)	0.234
Female	16 (20.51)	8 (27.59)	8 (16.33)	
Tumor length, mean ± SD	5.23 ± 1.71	6.04 ± 1.52	4.74 ± 1.64	**< 0.001**
cT stage, *n* (%)				
T_0_	3 (3.85)	0 (0.00)	3 (6.12)	**< 0.001**
T_1_	13 (16.67)	0 (0.00)	13 (26.53)	
T_2_	23 (29.49)	3 (10.34)	20 (40.82)	
T_3_	18 (23.08)	9 (31.03)	9 (18.37)	
T_4_	21 (26.92)	17 (58.62)	4 (8.16)	
cN stage, *n* (%)				
N_0_	14 (17.95)	0 (0.00)	14 (28.57)	**< 0.001**
N_1_	31 (39.74)	5 (17.24)	26 (53.06)	
N_2_	16 (20.51)	8 (27.59)	8 (16.33)	
N_3_	17 (21.79)	16 (55.17)	1 (2.04)	
Differentiated grade, *n* (%)				
Poorly	24 (30.77)	21 (72.41)	3 (6.12)	**< 0.001**
Moderate	35 (44.87)	7 (24.14)	28 (57.14)	
Well	19 (24.36)	1 (3.45)	18 (36.73)	
CA199, *n* (%)				
< 25 U/mL	68 (87.18)	23 (79.31)	45 (91.84)	0.212
≥ 25 U/mL	10 (12.82)	6 (20.69)	4 (8.16)	
CYF, *n* (%)				
< 3.3 ng/mL	62 (79.49)	16 (55.17)	46 (93.88)	**< 0.001**
≥ 3.3 ng/ml	16 (20.51)	13 (44.83)	3 (6.12)	

*Note:* Values are presented as no. (%), mean (± SD). The bold values highlight statistically significant results.

Abbreviations: (−), negative; (+), positive; CA199, carbohydrate antigen 199; cN stage, clinical N stage based on CT; cT stage, clinical T stage based on CT; CYF, cytokeratin fragment 21‐1; ESCC, esophageal squamous cell carcinoma; *p*, statistically significant; PNI, perineural invasion.

### 3.2 Characteristics of Spectral CT Parameters

**FIGURE 1:**
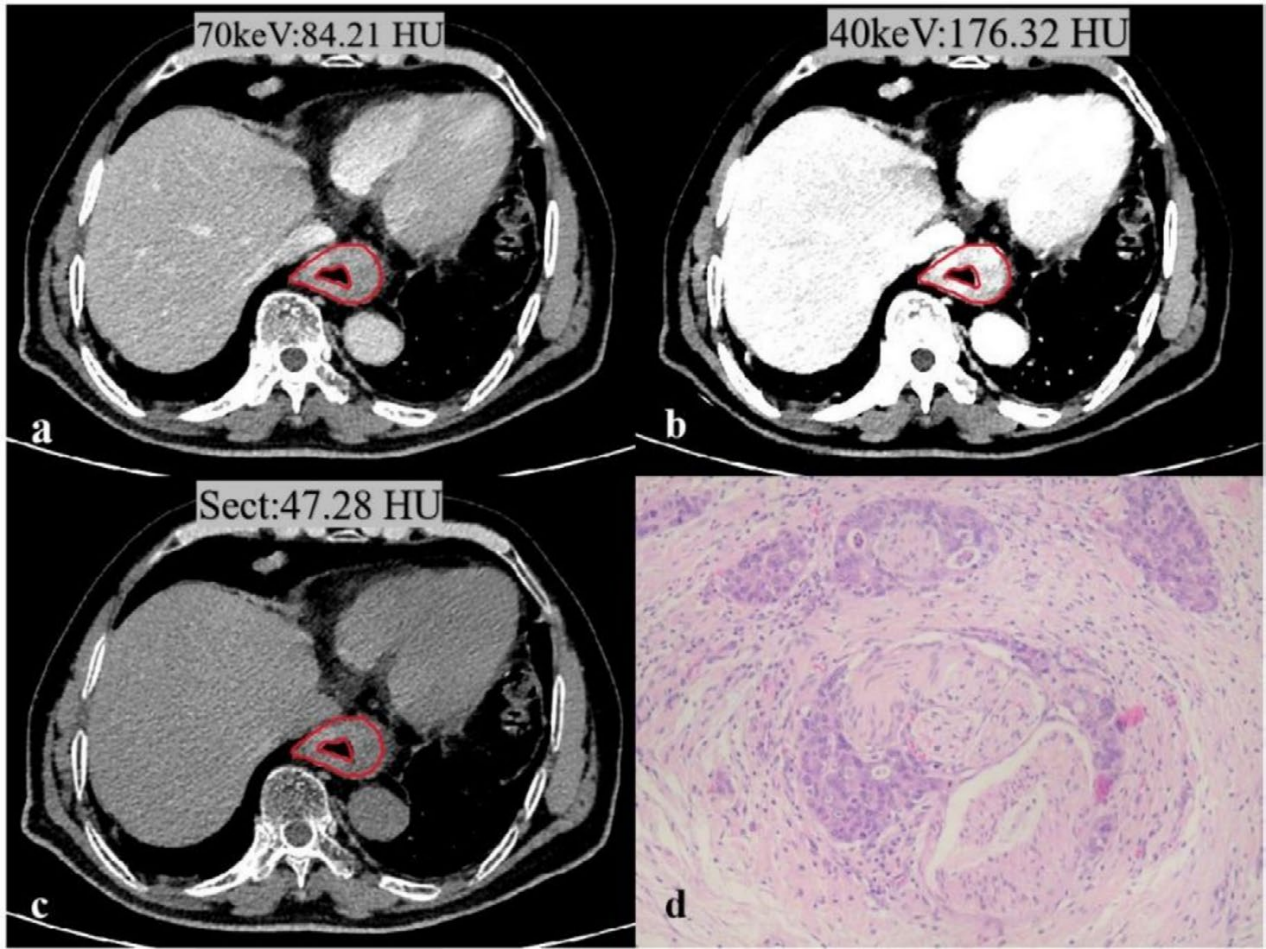
Spectral CT images obtained for a 63‐year‐old patient with PNI (+) in ESCC. (a–c) A tumor is located in the spectral CT images. (a) The 70 keV virtual monochromatic image; (b) The 40 keV virtual monochromatic image; (c) Conventional single‐energy CT; (d) The histopathological image (HE, magnification: 200×): ESCC invading the perineural space.

**FIGURE 2:**
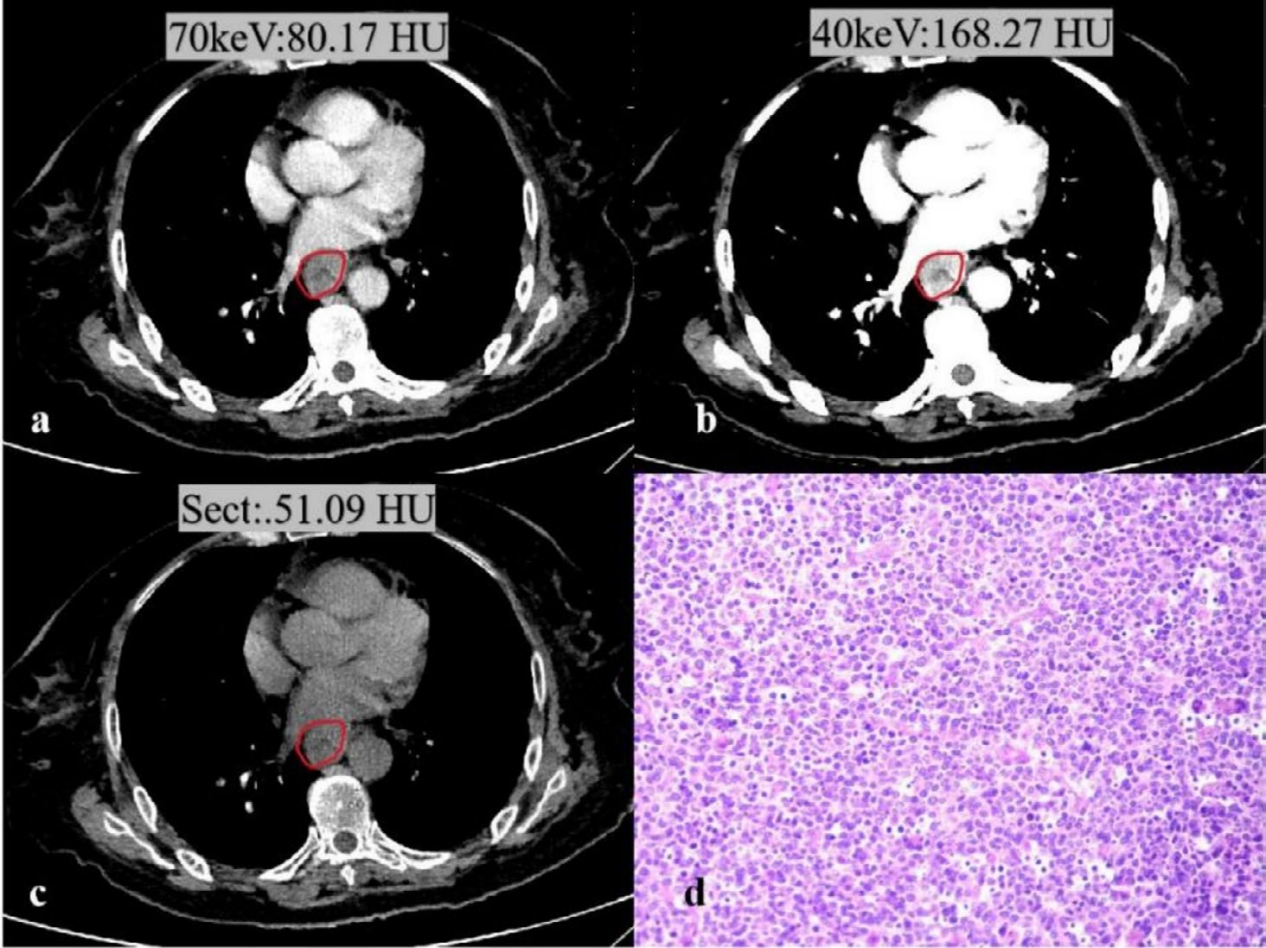
Spectral CT images obtained for a 67‐year‐old patient with PNI (−) in ESCC. (a–c) A tumor is located in the spectral CT images. (a) The 70 keV virtual monochromatic image; (b) The 40 keV virtual monochromatic image; (c) Conventional single‐energy CT; (d) The histopathology image (HE, magnification: 200×): ESCC.

Spectral CT parameters, including 40–70 keV attenuation, Zeff, and IC, were significantly higher in the PNI‐positive group than in the PNI‐negative group (all *p* < 0.05). However, Sect values were lower in the PNI‐positive group than in the PNI‐negative group (Table 2). The interobserver agreement for CT measurements was excellent, with ICC values ranging from 0.736 to 0.913. Representative PNI‐positive and PNI‐negative cases are shown in Figures 1 and 2.

**TABLE 2:** Spectral CT quantitative parameters in the evaluation of PNI status in ESCC patients.

Parameters	Overall	PNI		*p*
		Positive (+)	Negative (−)	
Sect, mean ± SD	49.30 ± 6.43	47.26 ± 6.29	50.51 ± 6.27	**0.030**
70 keV, mean ± SD	82.87 ± 9.01	89.51 ± 6.20	78.93 ± 8.08	**< 0.001**
60 keV, mean ± SD	98.63 ± 11.12	104.03 ± 8.66	95.43 ± 11.24	**< 0.001**
50 keV, mean ± SD	123.47 ± 16.49	132.64 ± 13.04	118.03 ± 15.99	**< 0.001**
40 keV, mean ± SD	158.03 ± 35.31	187.52 ± 15.41	140.58 ± 32.03	**< 0.001**
Zeff, mean ± SD	8.14 ± 0.25	8.25 ± 0.20	8.07 ± 0.26	**0.003**
IC, mean ± SD	1.31 ± 0.40	1.43 ± 0.51	1.24 ± 0.29	0.068

*Note:* Values are presented as no. (%), mean (± SD). The bold values highlight statistically significant results.

Abbreviations: (−), negative; (+), positive; ESCC, esophageal squamous cell carcinoma; IC, iodine concentration; *p*, statistically significant; PNI, perineural invasion; Sect, conventional single‐energy CT; Zeff, effective atomic number.

### 3.3 Construction of Nomogram Models

**FIGURE 3:**
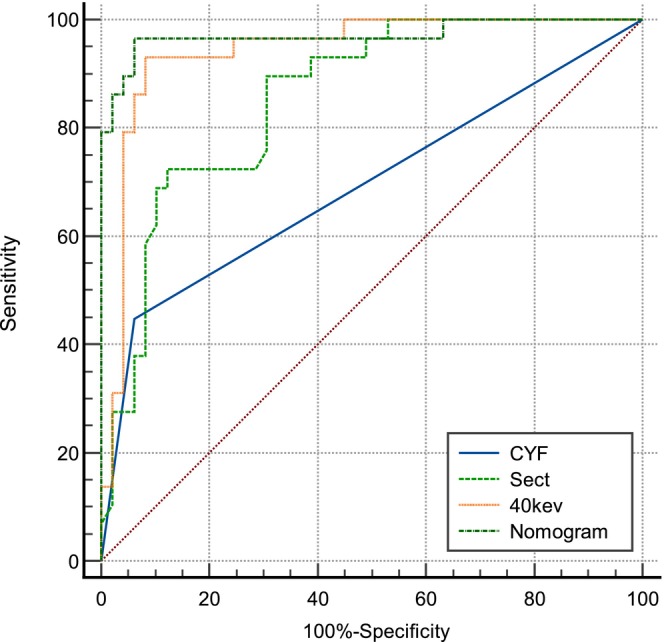
The ROC curves of clinical characterization parameters and nomogram in predicting PNI status.

Univariate and multivariate analyses identified CYF (*p* < 0.05), Sect (*p* < 0.05), and 40 keV (*p* < 0.05) as independent predictors of PNI status (Table 3). A nomogram incorporating these variables achieved high diagnostic performance (AUC = 0.971, 95% confidence interval [CI]: 0.906–0.996), outperforming individual parameters (Figure 3 and Table 4).

**TABLE 3:** Univariate and multivariate analysis of predictors of PNI.

Variables	Univariate^a^			Multivariate^a^		
	*β*	OR (95% CI)	*p*	*β*	OR (95% CI)	*p*
Age	−0.02	0.98 (0.94–1.03)	0.496			
Tumor length	0.50	1.65 (1.20–2.26)	**0.002**			
CA199	1.08	2.93 (0.75–11.45)	0.121			
CYF	2.52	12.46 (3.14–49.43)	**< 0.001**	3.10	22.21 (1.84–268.13)	**0.015**
Sect	−0.09	0.92 (0.84–0.99)	**0.036**	−0.25	0.78 (0.65–0.94)	**0.011**
70 keV	0.22	1.25 (1.12–1.38)	**< 0.001**			
60 keV	0.09	1.09 (1.03–1.16)	**0.002**			
50 keV	0.07	1.07 (1.03–1.12)	**< 0.001**			
40 keV	0.13	1.14 (1.07–1.21)	**< 0.001**	0.15	1.16 (1.07–1.26)	**< 0.001**
Zeff	3.92	50.52 (3.29–774.87)	**0.005**			
IC	1.28	3.59 (1.04–12.34)	**0.043**			

*Note:* Bold values highlight statistically significant results.

Abbreviations: CA199, carbohydrate antigen 199; CI, confidence interval; CYF, cytokeratin fragment 21‐1; IC, iodine concentration; OR, odds ratio; *p*, statistically significant; PNI, perineural invasion; Sect, conventional single‐energy CT; Zeff, effective atomic number.

^a^ Characteristics and parameters (*p* < 0.05) were included in univariate and multivariate analysis in turn.

**TABLE 4:** The diagnostic performance and DeLong test of spectral CT parameters and nomogram.

Parameters	AUC	95% CI	Sensitivity	Specificity	Accuracy	*p*
CYF	0.694	(0.595–0.792)	93.9%	44.8%	75.6%	< 0.001
Sect	0.857	(0.775–0.940)	87.8%	72.4%	82.1%	0.002
40 keV	0.943	(0.706–0.926)	91.8%	93.1%	92.3%	0.221
Nomogram	0.971	(0.906–0.996)	93.9%	96.6%	94.9%	—

Abbreviations: AUC, area under the ROC curve; CI, confidence interval; CYF, cytokeratin fragment 21‐1; *p*, statistically significant; Sect, conventional single‐energy CT.

### 3.4 Predictive Performance of Nomogram Models

Nomogram calibration curves demonstrated good agreement between predicted and actual PNI probabilities (Figure S2). Decision curve analysis confirmed the clinical utility of the models within a threshold probability range of 0.20–0.80 (Figure S3).

## 4 Discussion

**FIGURE 4:**
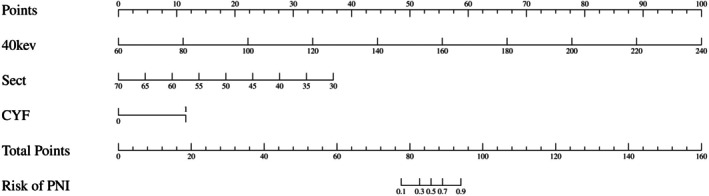
Nomograms for predicting perineural invasion (PNI) status in ESCC were developed using a binary logistic regression model incorporating CYF, Sect, and 40 keV attenuation values. Total points, calculated by summing individual factor scores, were plotted against the risk of PNI‐positive disease.

This study aims to investigate the value of spectral CT in predicting the status of PNI in patients with ESCC. We found that spectral CT parameters were remarkably different in distinguishing between PNI‐positive and PNI‐negative groups. We also discovered that multiple imaging features were associated with PNI status (Figure 4).

CYF, as a clinically actionable marker, provides valuable auxiliary information for predicting PNI status. In the analysis of CYF, a common tumor marker, CYF level was closely correlated with PNI status. In PNI‐positive patients, high CYF levels may reflect local tumor invasiveness, suggesting that tumors are likely to infiltrate the peripheral nerves [11, 12].

This study found a significant difference in pathological grading between the PNI‐positive and PNI‐negative groups (*p* < 0.05). The PNI‐positive group showed a higher proportion of poorly differentiated tumors, whereas moderately to well‐differentiated tumors were more common in the PNI‐negative group. These results suggest that less differentiated tumors possess greater invasive potential and are more likely to infiltrate surrounding neural tissues. Poorly differentiated tumors typically exhibit increased cellular atypia, higher proliferative activity, and reduced intercellular adhesion [13]. These biological features may facilitate invasion along nerve sheaths, contributing to the development and progression of PNI [14].

A significant difference was also found in cT and cN stages between the PNI‐positive and PNI‐negative groups (*p* < 0.05). The PNI‐positive group had a higher proportion of cT3–T4 and cN1–N2 diseases, indicating that PNI is more likely to occur with deeper local invasion and greater lymph node metastasis. T and N stages reflect the extent of local invasion and regional lymph node involvement, respectively [15]. As a specialized form of tumor invasion, PNI is often associated with such advanced disease characteristics, highlighting its value as an indicator of aggressive tumor behavior and poor prognosis [16].

We observed that Sect values in the PNI‐positive group were significantly lower than those in the PNI‐negative group. This statistically significant difference underscores the potential of Sect as a complementary predictor of PNI status. Low Sect values in tumors from patients with PNI‐positive disease may be closely related to alterations in vascular permeability or differences in the hydration status of tumors [17, 18, 19]. PNI‐positive tumors may exhibit reduced fat content or abnormal water distribution as a result of local inflammation or fibrosis. These changes, in turn, affect the distribution of fat and water on CT imaging, alterations that can directly lead to low Sect values [20].

In addition to CYF and Sect values, we investigated several energy‐spectrum CT parameters, including the 40 keV decay value, Zeff, and IC. Among these parameters, the elevated 40 keV decay value is particularly striking. This change may profoundly reflect the altered biological properties within the tumor tissue of PNI‐positive patients. Specifically, elevated 40 keV attenuation values might be closely related to increased cell density or altered blood flow patterns in tumor tissues [17, 21]. In the microenvironment of PNI‐positive tumors, cells may be tightly arranged due to accelerated proliferation, leading to an increase in cell density [22, 23]. At the same time, the development of tumor neovascularization and abnormalities in blood perfusion may affect the performance of attenuation values on CT images [24, 25]. These alterations in biological behavior not only reveal the aggressiveness of the tumor itself, but they may also produce the compression or infiltration of the peripheral nerves. This effect may consequently trigger PNI.

Univariate and multivariate analyses showed that the 40 keV attenuation value, Sect value, and CYF level were independent predictors of PNI status (*p* < 0.05). In particular, the combination of the 40 keV attenuation value, Sect value, and CYF level demonstrated a high predictive ability in the nomogram, with an AUC value of 0.971 (95% CI: 0.906–0.996), which was significantly higher than the AUC values of the other parameters of single‐energy spectral CT. This finding suggests that spectral CT parameters, especially the 40 keV attenuation value, can provide an efficient and accurate tool for the prediction of PNI status. The establishment of this line graph provides clinicians with a convenient and reliable predictive tool that can help determine the PNI status of patients with ESCC, thus providing a basis for individualized treatment.

Despite the positive results of this study regarding the application of spectral CT parameters, some limitations remain. First, the sample size was relatively small, which may limit the generalizability of the predictive model and increase the risk of overfitting. Future studies with larger, multicenter cohorts are needed to validate our findings. Second, the diagnosis of PNI mainly relies on pathological examination, and although spectral CT has demonstrated good performance in predicting PNI, its diagnostic value still needs to be verified by large, multicenter prospective studies. Furthermore, the current model is based on conventional spectral CT parameters; future studies could incorporate more advanced quantitative features such as texture analysis, radiomic signatures, or machine learning‐based integration of multiparametric data to further enhance predictive performance and support personalized treatment planning.

## 5 Conclusion

This study demonstrates the potential of spectral CT parameters, specifically 40 keV attenuation and Sect values, in predicting PNI status in ESCC. By combining these imaging parameters, the constructed column–line diagram provides clinicians with an effective predictive tool that is expected to play an important role in the development of individualized treatment plans. As studies advance in the future, spectral CT is expected to play an expanded role in the diagnosis and prognostic assessment of cancer.

## Author Contributions

**Zongbo Li:** conceptualization, methodology, software, data curation, investigation, validation, formal analysis, writing – original draft. **Wenzheng Lu:** conceptualization, methodology, writing – review and editing, software, formal analysis, investigation. **Yiheng Zhou:** conceptualization, methodology, validation. **Xiaofei Wu:** conceptualization, methodology, supervision. **Yuxi Ge:** conceptualization, methodology. **Wei Shao:** data curation, investigation. **Shudong Hu:** conceptualization, methodology, software, data curation, supervision, formal analysis, investigation, validation, funding acquisition, project administration, resources, writing – review and editing.

## Ethics Statement

The study was conducted in accordance with the 1964 Helsinki Declaration and approved by the Institutional Review Board of Affiliated Hospital of Jiangnan University (LS2024589).

## Consent

Patient consent was waived since it was a retrospective study.

## Conflicts of Interest

The authors declare no conflicts of interest.

## Supporting information


**Figure S1:** Flowchart of the patient selection pathway.
**Figure S2:** The calibration curve of nomogram. The abscissa represents the predicted value, and the ordinate represents the actual value. The gray diagonal line serves as a reference line. If the black curve is close to the reference line, it means that the predicted value and the actual value are more consistent.
**Figure S3:** Decision curve analysis of nomogram. The ordinate represents net income, the first abscissa represents the threshold probability, and the second abscissa represents the profit‐loss ratio. “None” represents a horizontal line, which means that all samples are judged as negative, “All” represents a slope, which means that all samples are judged as positive, and the Nomogram curve is the curve we care about. Within the threshold probability range of 0.1–0.8, the Nomogram curve is located above the two baselines of “None” and “All”, which indicates that the performance of the model is acceptable within this range.

## Data Availability

Data will be made available on reasonable request.
