# Supplementary material for: Preoperative Diagnostic Value of Spectral CT for Predicting Perineural Invasion in Esophageal Cancer
Source: Cancer Med. 2025 Nov 20;14(22):e71403. doi: 10.1002/cam4.71403 (PMC12631740; doi:10.1002/cam4.71403)
Supplement: Supplementary file 1 — Figure S1: Flowchart of the patient selection pathway. Figure S2: The calibration curve of nomogram. The abscissa represents the predicted value, and the ordinate represents the actual value. The gray diagonal line serves as a reference line. If the black curve is close to the reference line, it means that the predicted value and the actual value are more consistent. Figure S3: Decision curve analysis of nomogram. The ordinate represents net income, the first abscissa represents the threshold probability, and the second abscissa represents the profit‐loss ratio. “None” represents a horizontal line, which means that all samples are judged as negative, “All” represents a slope, which means that all samples are judged as positive, and the Nomogram curve is the curve we care about. Within the threshold probability range of 0.1–0.8, the Nomogram curve is located above the two baselines of “None” and “All”, which indicates that the performance of the model is acceptable within this range. [file CAM4-14-e71403-s001.docx]

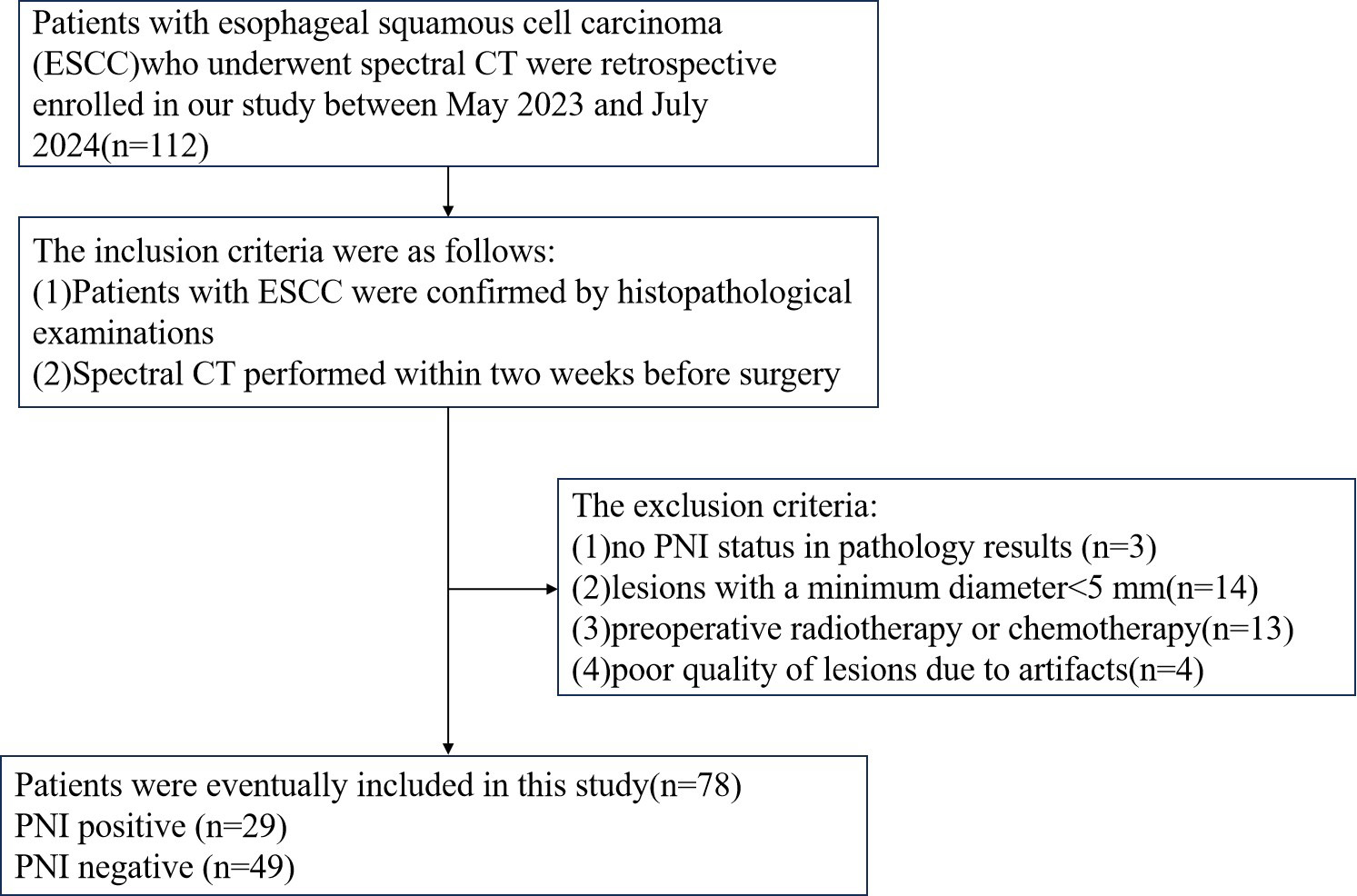


**Supplementary Fig. S1** Flowchart of the patient selection pathway.


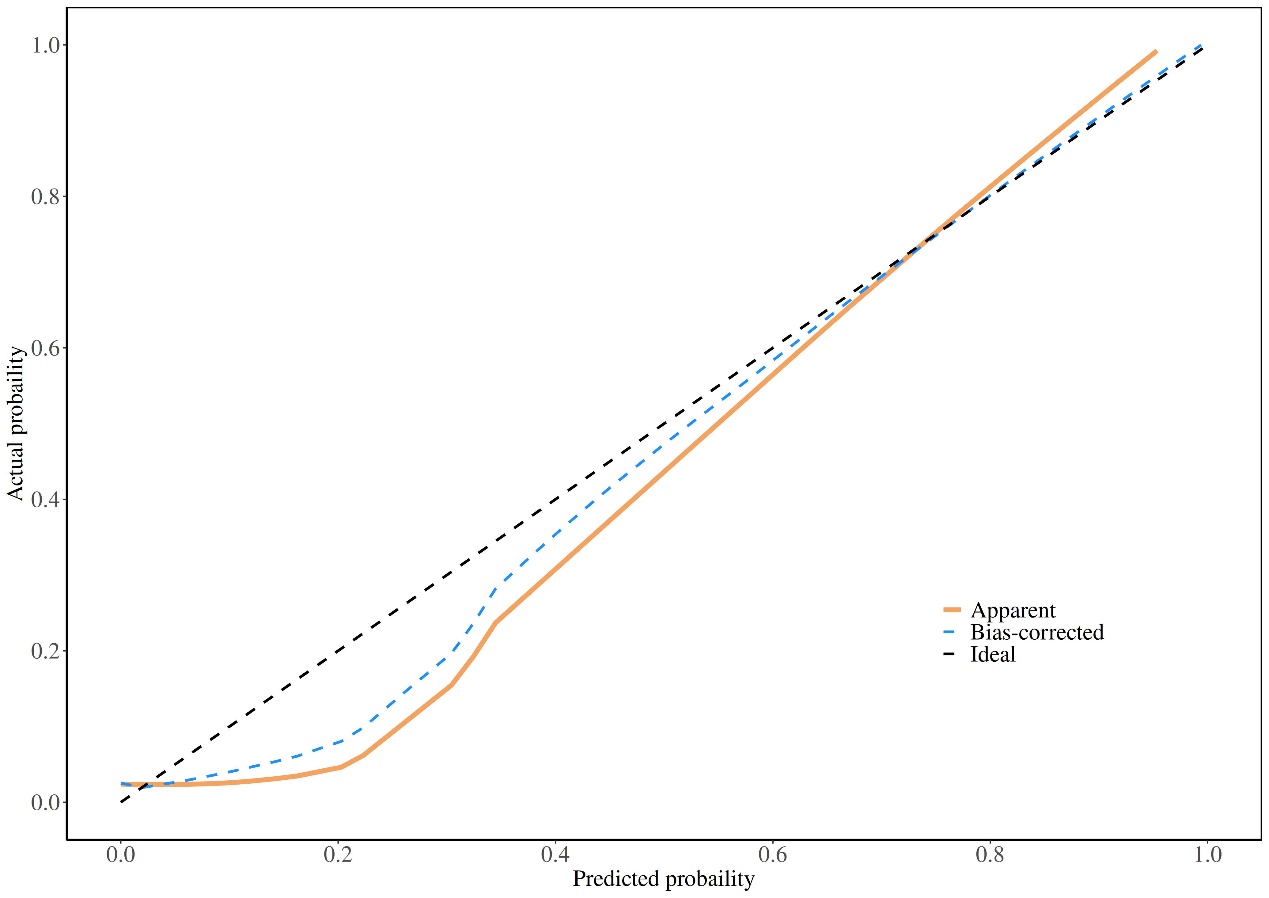


**Supplementary Fig. S2** The calibration curve of nomogram. The abscissa represents the predicted value, and the ordinate represents the actual value. The gray diagonal line serves as a reference line. If the black curve is close to the reference line, it means that the predicted value and the actual value are more consistent.


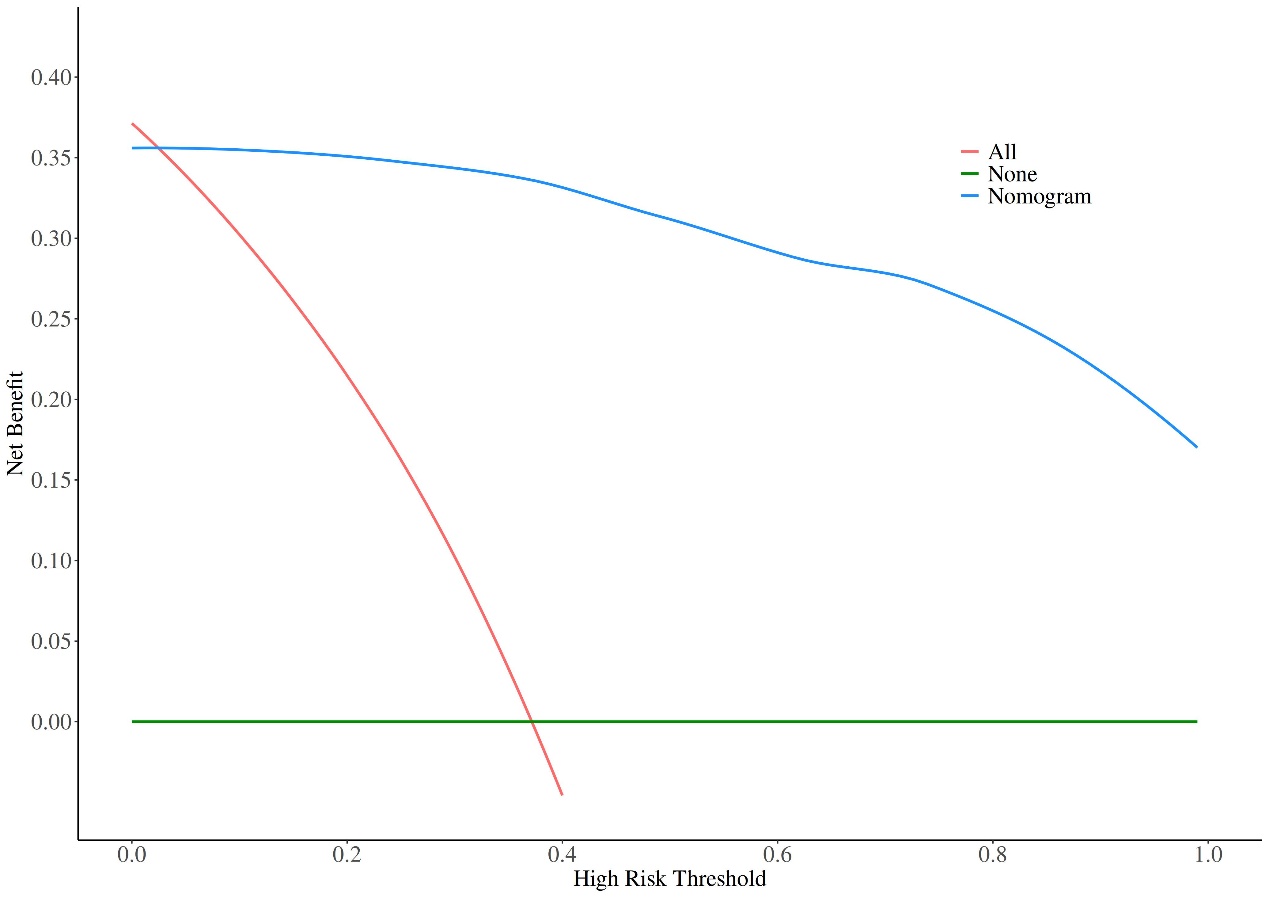


**Supplementary Fig. S3** Decision curve analysis of nomogram. The ordinate represents net income, the first abscissa represents the threshold probability, and the second abscissa represents the profit-loss ratio. "None" represents a horizontal line, which means that all samples are judged as negative, "All" represents a slope, which means that all samples are judged as positive, and the Nomogram curve is the curve we care about. Within the threshold probability range of 0.1 to 0.8, the Nomogram curve is located above the two baselines of "None" and "All", which indicates that the performance of the model is acceptable within this range.
